# The ERK MAP kinase-PEA3/ETV4-MMP-1 axis is operative in oesophageal adenocarcinoma

**DOI:** 10.1186/1476-4598-9-313

**Published:** 2010-12-09

**Authors:** Richard Keld, Baoqiang Guo, Paul Downey, Christian Gulmann, Yeng S Ang, Andrew D Sharrocks

**Affiliations:** 1 Faculty of Life Sciences, University of Manchester, Michael Smith Building, Oxford Road, Manchester, M13 9PT, UK; 2 Faculty of Medicine, University of Manchester, Oxford Road, Manchester, UK; 3 Department of Histopathology, Beaumont Hospital, Dublin, Ireland

## Abstract

**Background:**

Many members of the ETS-domain transcription factor family are important drivers of tumourigenesis. In this context, their activation by Ras-ERK pathway signaling is particularly relevant to the tumourigenic properties of many ETS-domain transcription factors. The PEA3 subfamily of ETS-domain transcription factors have been implicated in tumour metastasis in several different cancers.

**Results:**

Here, we have studied the expression of the PEA3 subfamily members PEA3/ETV4 and ER81/ETV1 in oesophageal adenocarcinomas and determined their role in oesophageal adenocarcinoma cell function. PEA3 plays an important role in controlling both the proliferation and invasive properties of OE33 oesophageal adenocarcinoma cells. A key target gene is *MMP-1*. The ERK MAP kinase pathway activates PEA3 subfamily members and also plays a role in these PEA3 controlled events, establishing the ERK-PEA3-MMP-1 axis as important in OE33 cells. PEA3 subfamily members are upregulated in human adenocarcinomas and expression correlates with *MMP-1 *expression and late stage metastatic disease. Enhanced ERK signaling is also more prevalent in late stage oesophageal adenocarcinomas.

**Conclusions:**

This study shows that the ERK-PEA3-MMP-1 axis is upregulated in oesophageal adenocarcinoma cells and is a potentially important driver of the metastatic progression of oesophageal adenocarcinomas.

## Introduction

Oesophageal adenocarcinoma is a devastating disease that has been rising year on year over the past three decades and is the 6^th ^highest cause of cancer mortality in the UK, accounting for around 5% of all cancers [1,2]. The escalating incidence is thought to be a result of the combination of an obesity epidemic, an aging population, and *H. pylori *eradication [3-5]. The disease is curable by surgery or endoscopic therapy if diagnosed at a very early stage [6] but usually, diagnosis is made at an advanced stage with the presence of lymph node and distant metastases [5]. There are few clear prognostic indicators of susceptibility to developing oesophageal adenocarcinoma although patients with Barrett's oesophagus are thought to be more at risk to developing oesophageal adenocarcinoma. However, the progression from Barrett's oesophagus to dysplasia and subsequent adenocarcinoma is unpredictable and poorly understood [7]. The lack of prognostic indicators results in presentation of patents at late disease stages, resulting in poor five year survival rates and patients usually succumb to disease re-occurrence [5,8]. For a significant majority, surgery is not beneficial and in such patients with distant metastases, survival is limited to 9 months [9-11]. If the situation is to change then a deeper understanding of tumour growth and metastases is needed to identify new treatment targets.

The ETS domain transcription factor family consists of a group of 27 proteins in humans that all contain the conserved ETS DNA-binding domain and share a core DNA binding specificity centred around the sequence GGA^A^/_T _[12,13]. The PEA3 subfamily includes three transcription factors, PEA3 (also known as ETV4 and E1AF), ER81 (also known as ETV1) and ERM (also known as ETV5). These proteins all contain three conserved domains with sequence identity of 95%, 85% and 50% in the ETS, acidic and Ct domains respectively [14]. This similarity potentially allows for an overlap in PEA3 subfamily function through acting on a common set of target gene promoters. Indeed due to their conserved DNA binding domain, significant overlap in promoter binding has been observed more generally amongst ETS domain transcription factors [15,16]. The PEA3 subfamily plays an important role in embryogenesis, especially in neurogenesis [17] and also in mammary gland development [14,18,19]. In the adult, PEA3 subfamily members are generally expressed at lower levels and in a more restrictive manner [14] but ETS domain proteins, and especially the PEA3 subfamily are associated with carcinogenesis, especially tumour metastases and their overexpression often indicates adverse prognosis [14,20]. This has been shown to be the case in breast cancer, colon cancer, ovarian cancer and gastric cancer [14]. More recently, high expression levels of ER81 have been shown to occur in prostate cancer as a result of chromosomal translocations of the ER81 gene into loci with high promoter activity in prostate cells [21,22]. PEA3 expression often correlates with enhanced invasive properties and hence is associated with metastasis. For example, in gastric cancer and colon cancer cells, PEA3 inhibition reduces cell invasion *in vitro *[23,24]. Conversely, PEA3 over-expression induces an invasive phenotype in breast and ovarian cancer cells [25,26]. Similarly ER81 over-expression enhances the invasive capabilities of prostate cancer cells [22]. The invasive phenotypes of cells with high PEA3 subfamily expression are thought to be due in part to their ability to regulate the expression of matrix metalloproteases (MMPs) [20]. MMP1 has been shown to be an adverse marker in oesophageal adeoncarcinoma [27,28]. In colon and gastric cancer cell lines, PEA3 has been shown to regulate *MMP-1 *and *MMP-7 *expression [23,24]. A potential link between PEA3 and MMP7 expression was also suggested in studies on oesophageal squamous carcinoma cells [29]. MAP kinase signalling is also important in PEA3 activation [30,31] in part through driving its dynamic sumoylation [32]. Importantly MAP kinase signaling synergises with PEA3 in *MMP *activation as demonstrated by enhanced MMP-9 and MMP-14 production in response to EGFR signaling in ovarian cancer [25]. These observations indicate that PEA3 subfamily members are likely central regulators in carcinogenesis and are potential therapeutic targets.

A unifying view of PEA3 function in cancer is therefore that it is a regulator of *MMP *expression in response to ERK MAP kinase pathway signaling. However, to date few studies have connected these molecular events together in a single system and the potential role of PEA3 subfamily members in oesophageal adenocarcinoma has not previously been investigated. Indeed, none of the wider ETS domain transcription factor family has been implicated in oesophageal adenocarcinoma, although Ets-1, Ets-2 and Elk-1 have been shown to be over-expressed on squamous oesophageal cancers [33-35]. Here, we show that high PEA3 expression is a frequent occurrence in oesophageal adenocarcinoma. In oesophageal adenocarcinoma cell line models, PEA3 plays a role in promoting invasion and is also important for oesophageal cell proliferation. Molecularly, the invasive properties are likely due to the activation of *MMP-1 *expression. Furthermore we also show an important role of the ERK pathway in promoting PEA3 activity and ensuing invasion. In adenocarcinoma tissue, the co-occurrence of PEA3 family member expression correlates with enhanced *MMP-1 *expression. Active ERK signaling correlates with enhanced stage suggesting an important role in promoting metastasis via PEA3 and ER81. These results indicate that the ERK-PEA3-MMP-1 axis identified in oesophageal cancer cells is also likely to be operative in oesophageal adenocarcinoma tissue. This pathway could potentially be targeted by drug inhibition with a view to improve prognosis.

## Results

### The expression of PEA3 family members in oesophageal tissues

**Figure 1:**
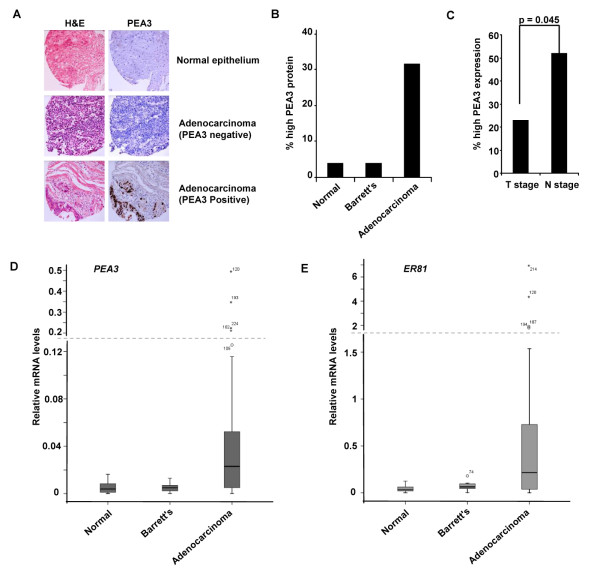
**PEA3 and ER81 mRNA and protein expression in oesophageal adenocarcinomas**. (A) Examples of TMA staining for PEA3 protein (brown stain in right hand panels) in normal epithelium and adenocarcinoma samples at × 20 magnification. H&E staining of the same samples is shown on the left. (B) Summary of TMA data for moderate-high PEA3 expression in patient samples from different tissue classes. (C) The proportion of patients with T stage or N stage disease, with high PEA3 protein expression. (D and E) Box plots of *PEA3 *(D) and *ER81 *(E) mRNA expression in oesophageal tissue taken from healthy controls, Barrett's oesophagus and oesophageal adenocarcinoma patients. Median relative expression levels of *PEA3 *and *ER81 *are indicated for each tissue type. mRNA expression is calculated relative to 18S ribosomal RNA. The box plot represents the inter-quartile range and the median value is indicate by the horizontal line. The y axes are split (indicated by dashed lines) and the high outliers are labelled by case number.

To establish whether members of the PEA3 subfamily ETS-domain transcription factors might play a role in oesophageal adenocarcinomas, we first determined the expression of PEA3 protein in normal oesophageal tissue and oesophageal adenocarcinomas by constructing a TMA from 27 samples from normal patients and 58 samples from oesophageal adenocarcinomas, along with samples from adjacent normal tissue. We also included 23 samples from patients with Barrett's oesophagous as this is thought to be a precursor condition to adenocarcinoma development [7]. Samples were then scored as PEA3 positive if they had moderate-high PEA3 protein levels (Figure 1A). Very few normal or Barrett's samples contained moderate-high PEA3 protein levels (4%) but in contrast, over 33% of samples from adenocarcinomas exhibited moderate-high PEA3 protein levels (Figure 1B). Importantly, when we split the adenocarcinomas into T and N stage tumours, the frequency of occurrence of high PEA3 protein levels was significantly higher in the nodal (N stage) tumours, suggesting an association of PEA3 expression with metastasis (Figure 1C).

In addition to analysing protein levels, we also determined the levels of *PEA3 *mRNA in oesophageal tissue samples alongside the levels of the related subfamily member *ER81*. The levels of *PEA3 *and *ER81 *mRNA were generally low in samples from normal tissue or Barrett's patients (Additional file 1: Figure S1; Figure 1D and 1E; see also Figure 6 A). In contrast, samples from oesophageal adenocarcinomas generally showed higher levels of either *PEA3*, *ER81 *or both transcription factors (Additional file 1: Figure S1; 1D and 1E; see also Figure 6 A). Indeed of the 38 adenocarcinomas analysed, 29 (79%) showed levels of either *PEA3 *or *ER81*, or both, that were higher than found in samples from normal tissue.

Together these data therefore provide strong evidence which associates *PEA3 *and *ER81 *expression with adenocarcinomas, and association with patient parameters suggests that PEA3 expression is associated with metastatic disease.

### The expression of PEA3 family members and their target genes in oesophageal cell lines

**Figure 2:**
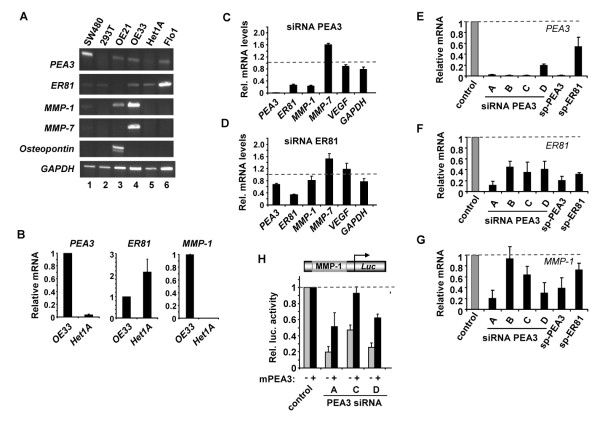
**PEA3 expression and *MMP-1 *regulation**. (A) RT-PCR analysis of *PEA3, ER81, MMP-1, MMP-7 *and *osteopontin *mRNA expression in the indicated cell lines. *GAPDH *was used as a loading control. (B-D) Real time RT-PCR analysis of *PEA3, ER81, MMP-1 *and the indicated putative target genes in untreated Het1A and OE33 cells (B) or OE33 oesophageal adenocarcinoma cells treated with SMARTpool siRNAs directed against PEA3 (C) or ER81 (D). Average mRNA levels (from duplicate samples in 2 experiments) were calculated relative to the respective mRNA levels of each gene in OE33 cells (B) or to cells treated with a non targeting siRNA (C and D) (taken as 1). (E-G) Real time RT-PCR analysis of *PEA3 *(E), *ER81 *(F) and *MMP-1 *(G) expression in OE33 cells treated with SMARTpool (sp) siRNAs directed against PEA3 or ER81 or one of four individual de-convoluted SMARTpool component siRNAs against PEA3, denoted A-D. Average mRNA expression levels (from duplicate samples in 2 experiments) were calculated relative to the respective mRNA levels of each gene in cells treated with a non targeting siRNA (taken as 1). (H) Reporter gene assay of the activity of a MMP-1-luciferase reporter construct in OE33 cells in the presence of the indicated siRNA duplexes. Cells were co-transfected with either empty vector (grey bars) or with a murine PEA3 expression vector (black bars). Data are shown relative to the activity of the reporter in the presence of non-targeting siRNA duplexes in either the presence or absence of mPEA3 (taken as 1 in both cases) and are the average of 2 experiments performed in duplicate (+/- sem).

Next we investigated whether oesophageal cell lines showed similar characteristics to the tumour samples. Two cell lines derived from oesophageal adenocarcinomas, Flo-1 and OE33 cells were tested alongside OE21 oesophageal squamous cancer cells, and Het1A, a cell line derived from normal oesophageal epithelial tissue. SW480 and 293T cells were used as controls as these have previously been shown to be positive and negative respectively for PEA3 expression [23,36]. Both of the adenocarcinoma cell lines showed detectable *PEA3 *mRNA expression whereas normal Het1A cells showed little expression (Figure 2A, panel 1, lanes 3-5). Low levels of ER81 mRNA were seen in all cell lines, except OE21 where it was barely detectable and Flo1 cells where high level expression was observed (Figure 2A, panel 2). These results were confirmed in OE33 and Het1A cells by real-time PCR, where *PEA3 *levels are clearly greatly elevated in OE33 cells (Figure 2B). OE33 and Het1A cells therefore represent reasonable models in which to study PEA3 function as PEA3 expression mirrors that seen in tissue samples, being high in adenocarcinomas and low in normal oesophageal cells.

PEA3 has been shown to control the expression of several matrix metalloproteases, including *MMP-1 *[23,36] and *MMP-7 *[24], and other genes such as *osteopontin *[37] and *VEGF *[38]. We therefore examined whether PEA3 presence correlated with expression of any of these potential targets in the cell line models. *MMP-1 *was expressed in both OE21 and OE33 cell lines, alongside PEA3 suggesting a causal relationship (Figure 2A, panel 3, lanes 3 and 4). These results were confirmed in OE33 and Het1A cells by real-time PCR, where *MMP-1 *levels are clearly greatly elevated in OE33 cells (Figure 2B). In contrast *MMP-7 *was only expressed to high levels in OE33 cells and reciprocally, *osteopontin *was only expressed to high levels in OE21 cells (Figure 2A, panel 4, lanes 3 and 4). Flo1 cells showed little *MMP *expression despite the presence of *PEA3 *and *ER81*, indicating that these transcription factors are not sufficient to activate MMP expression.

To further investigate the potential links between PEA3 and ER81 and putative target gene expression, we performed siRNA-mediated depletion experiments in OE33 cells using SMARTpools and measured target gene expression. Depletion of PEA3 had little effect on *GAPDH *and *VEGF *levels, but caused a 75% reduction in *MMP-1 *mRNA expression (Figure 2C). A moderate 1.6 fold rise in *MMP-7 *levels was observed upon PEA3 depletion (Figure 2C). In contrast, depletion of ER81 had minimal effects on potential target gene expression, although the incomplete levels of knockdown seen with ER81 (65% reductions; Figure 2D) might mask potential effects which would be revealed by complete knockdown. Interestingly, *ER81 *levels were reduced upon PEA3 depletion (Figure 2C) and reciprocally, *PEA3 *levels were reduced upon *ER81 *depletion, although to a lesser extent, (Figure 2D) suggesting potential cross-regulation (see discussion). To verify these results, we deconvoluted the PEA3 SMARTpool siRNAs and analysed the effects on *MMP-1 *expression. First we confirmed that the individual siRNAs caused *PEA3 *depletion, and all showed efficient depletion of *PEA3 *levels (78-99% reductions; Figure 2E) but also impacted on *ER81 *levels, albeit to a lesser extent (64-95% reductions; Figure 2F). Importantly, three of the four individual siRNA constructs also caused reductions in *MMP-1 *levels (Figure 2G) with the exception of siRNA-B which presumably triggers a compensatory off target effect. To confirm the specificity of the siRNA effects, we performed a rescue experiment with murine PEA3 expression constructs. siRNA constructs A, C and D all caused similar reductions in the activity of a *MMP-1 *promoter-driven reporter construct to those observed on the expression of the endogenous gene (Figure 2H). Re-introduction of wild-type PEA3 protein, caused a reversal of the siRNA effects, demonstrating that the loss of PEA3 was at least in part responsible for the reduced *MMP-1 *levels observed. However, as PEA3 depletion also results in decreased ER81 levels, we cannot definitively conclude that PEA3 is directly responsible for all of the downstream effects on *MMP-1 *expression and cell behaviour, although it is clearly a major contributory factor.

Together these results therefore establish OE33 cells as a useful model to study PEA3 function in adenocarcinoma cells as they express both *PEA3*, and its target gene *MMP-1*. Furthermore PEA3 is necessary for *MMP-1 *expression in these cells. Importantly PEA3 family expression is not sufficient for MMP expression in all cell lines as *MMP-1 *and *-7 *are not highly expressed in Flo1 cells despite the expression of these transcription factors.

### Comparative analysis of oesophageal cell phenotypes

**Figure 3:**
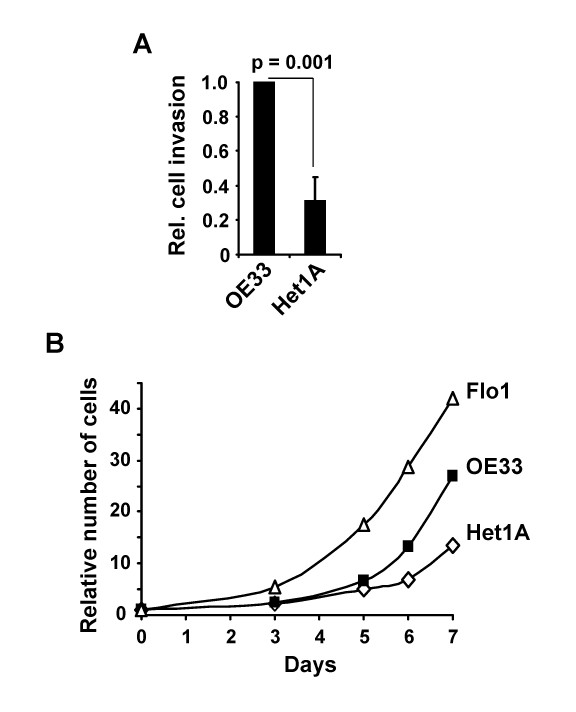
**Proliferative and invasive properties of oesophageal cells**. (A) Relative number of OE33 and Het1A cells invading through a 8 μm matrigel chamber. The data are presented relative to the number of invading OE33 cells. Data are the mean and standard deviations of the relative number of invading cells from 3 experiments. Statistical significance was tested by the t test. (B) Comparative analysis of the relative number of adherent Flo1, OE33 and Het1A, cells grown for 7 days. 2 × 10^4 ^cells were seeded at day 0 (indicated as 1). The data are representative of two independent experiments.

We have demonstrated that the gene expression profiles of the OE33 oesophageal adenocarcinoma cells differ from Het1A oesophageal epithelial cells and we wanted to know if the phenotypes of these cell lines also differed. First we used Matrigel invasion chambers to assess the capacity of these cells to migrate and invade *in vitro*. OE33 cells displayed a 3 fold increase in invasive potential when compared to Het1A cells (Figure 3A). This difference is consistent with the higher *MMP-1 *expression seen in OE33 cells, as MMP-1 is often associated with metastatic-like invasive properties.

Next we compared the proliferation of several oesophageal cell lines by counting the cells over a 7 day period. Het1A cells were compared to OE33 and Flo-1 cells. All of the cell lines proliferate exponentially. However the OE33 and Flo-1 adenocarcinoma-derived cells proliferate quicker than the Het1A cells (Figure 3B). Similar levels of cell death were seen in all cases, indicating that increased survival was not responsible for the higher numbers of cells observed with the adenocarcinoma cell lines (data not shown).

Together, these results establish that OE33 adenocarcinoma cells exhibit a higher invasive potential and growth rate than the non tumourigenic Het1A cells.

### PEA3 is required for the increased invasion and proliferation in OE33 cells

**Figure 4:**
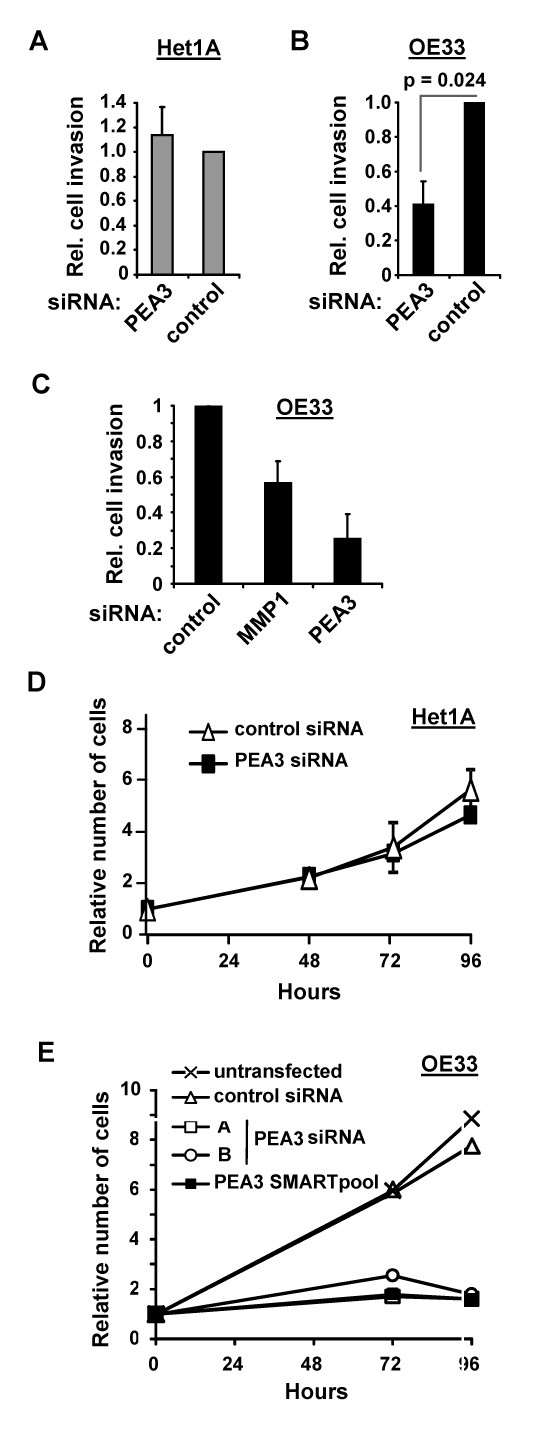
**PEA3 controls the proliferation and invasion of OE33 oesophageal adenocarcinoma cells**. (A-C) Invasion assays of Het1A (A) and OE33 (B and C) cells in the presence of siRNA directed against PEA3, MMP-1 or a non targeting siRNA (control). Assays were performed for 24 hours and the number of invading cells was compared to the average number of invading cells treated with a non targeting control (taken as 1). Data are the mean and standard deviations of the relative number of invading cells from duplicate samples in 2-3 independent experiments. Statistical significance was tested by the t test. (D and E) Comparative analysis of the relative number of adherent Het1A (D) and OE33 (E) cells grown for 96 hours in the presence of the indicated targeting or non-targeting (control) siRNA duplexes. SMARTpool siRNAs (D and E) and individual siRNA contructs A and B (E) against PEA3 were used. 2 × 10^4 ^cells were seeded at day 0 (indicated as 1). The data are representative of three independent experiments, and in (D) show the mean relative cell numbers and standard deviations from two experiments.

PEA3 has been established as an important regulator of cell invasion in colon cancer and gastric adenocarcinoma cells through regulation of *MMP-1 *and *MMP-7 *respectively [23,24]. We therefore wanted to investigate if PEA3 is also a regulator of oesophageal cancer cell invasion. A siRNA-mediated PEA3 knockdown strategy was employed to reduce PEA3 expression. Matrigel invasion chambers were again utilised to assess *in vitro *invasion. Het1A cells do not express PEA3 at high levels making them a valid control for PEA3 depletion. Indeed, depletion of PEA3 did not alter Het1A cell invasion when compared to cells treated with control duplexes (Figure 4A). This indicates that the PEA3 SMARTpool is unlikely to have an 'off target' effect on cell invasion. In contrast, PEA3 depletion reduced the invasive capabilities of OE33 by nearly 60% (Figure 4B), indicating that PEA3 is important for invasion by OE33 cells. To further extend our link between PEA3, MMP-1 and invasion, we asked whether MMP-1 depletion in OE33 cells would also lead to a decrease in invasion. This was indeed the case, albeit to a lesser extent (Figure 4C), suggesting that PEA3 likely drives invasion through multiple targets in addition to MMP-1.

Research on PEA3 has mainly focused on its ability to regulate MMPs and cell invasion. A previous studies in breast and ovarian cancer cells demonstrated that PEA3 controls the expression of cell cycle regulators such as *Cyclin D3 *[39] and *p21 *[40] respectively, and hence suggested that it might be involved in controlling proliferation. We therefore investigated if PEA3 was important for oesophageal cancer cell proliferation. First we depleted PEA3 in Het1A cells. Over a 96 hour period, the proliferation of Het1A cells was similar to cells treated with control duplexes (Figure 4D). In contrast, OE33 cells treated with either SMARTpool siRNA against PEA3 or the deconvoluted siRNA constructs A and B, exhibited a sustained a growth arrest (Figure 4E).

In summary, PEA3 is required for the proliferation and enhanced invasive properties of OE33 adenocarcinoma cells.

### ERK MAP kinase signalling is important for OE33 cell proliferation and invasion

**Figure 5:**
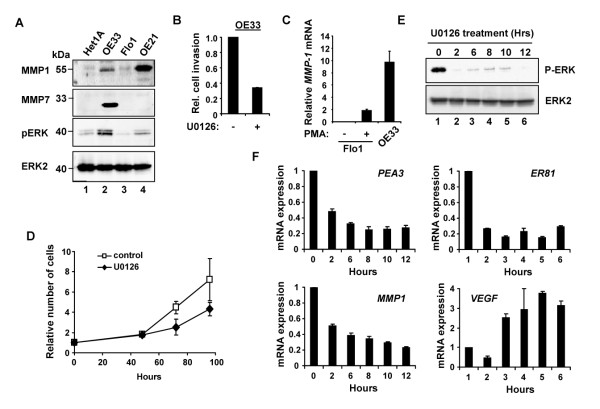
**Role of ERK MAP kinase signaling in OE33 cell function**. (A) Western blot of MMP-1, MMP-7 and phospho-ERK in the indicated oesophageal cell line extracts. ERK2 is used as a loading control. The positions of molecular weight markers are indicated. (B) Invasion assays of OE33 cells in the presence of U0126 or DMSO (-). Assays were performed for 48 hours and the number of invading cells was compared to the average number of invading cells treated with DMSO (taken as 1). Data are the mean and standard deviations of the relative number of invading cells from 2-3 experiments performed in duplicate. (C) Real time RT-PCR analysis of *MMP-1 *expression in untreated OE33 cells or Flo1 cells treated or untreated with PMA (average of duplicate samples in 2 experiments). (D) Comparative analysis of the relative number of adherent and OE33 cells grown for 96 hours in the presence of U0126 or DMSO (control). 2 × 10^4 ^cells were seeded at day 0 (indicated as 1). The data are the mean relative cell numbers and standard deviations from two independent experiments. (E) Western blot of OE33 cell extracts for phospho-ERK. The cells were treated with U0126 at time 0 and allowed to grow for 12 hours. ERK2 was used as a loading control. (F) Real time RT-PCR analysis of *PEA3, ER81*, *MMP1 *and *VEGF *expression in OE33 cells treated with U0126 for the indicated time periods (0-12 hr). Average mRNA levels (from duplicate samples) were calculated relative to the respective mRNA levels of each gene in cells at time zero (taken as 1).

Previous studies have demonstrated that PEA3 activity is potentiated by ERK MAP kinase pathway signalling [14] and that this signalling pathway plays an important role in cancer cell properties, including invasion and proliferation [41]. We therefore investigated the activation status of this pathway in oesophageal-derived cell lines by western analysis using an anti-phospho-ERK antibody. Amongst the four lines studied, phospho-ERK levels were highest in OE33 cells, indicating that the ERK pathway is active in these cells (Figure 5A, lane 2). OE33 cells also contained high levels of MMP-1 and MMP-7 protein, which is consistent with their relative mRNA expression levels (Figure 5A, top two panels lane 2; Figure 2A, lane 4). However, there appears to be additional post-transcriptional events acting on MMP-1 as OE21 show more MMP-1 protein than OE33 cells yet contain less *MMP-1 *mRNA (Figure 5A, top panel, lanes 2 and 4; Figure 2A, lanes 3 and 4). In contrast, Flo1 cells contained little MMP-1 mRNA or protein and very low levels of phospho-ERK (Figure 5A, lane 4). Thus the lack of ERK signaling in these cells likely explains why MMPs are not highly expressed despite the presence of PEA3 family members. To test this hypothesis, we treated Flo1 cells with PMA to activate ERK pathway signalling. A substantial increase in *MMP-1 *expression was observed (Figure 5C), in keeping with the idea that ERK pathway signalling is required for MMP-1 induction in addition to PEA3 overexpression.

Having established that ERK signalling levels were high in OE33 cells we used the MEK inhibitor U0126 to block ERK signalling and investigated its effect on OE33 cell invasion and proliferation. Both invasion (Figure 5B) and proliferation (Figure 5D) of OE33 cells were severely impaired upon inhibition of the ERK pathway. Finally, we investigated whether ERK signalling impacted on the activity of the PEA3 target gene *MMP-1*. Treatment of OE33 cells with U0126 effectively reduced ERK activation over a sustained period (Figure 5E). Importantly, *MMP-1 *expression levels were also reduced (Figure 5F), consistent with the known connections between ERK pathway signalling and PEA3-mediated gene expression. We also observed a decrease in the expression of both *PEA3 *and *ER81 *levels upon U0126 treatment, indicating a role for ERK pathway signalling in maintaining their expression (Figure 5F). However, generic effects on gene expression were not observed as *VEGF *was only transiently inhibited, and then superinduced, suggesting regulation by alternative mechanisms (Figure 5F).

Together, these results reveal that ERK pathway activity is elevated in OE33 adenocarcinoma cells, and plays an important role in invasion, proliferation and the regulation of PEA3-associated gene expression.

### *MMP-1/-7 *expression and ERK pathway signalling status in oesophageal tissue specimens

**Figure 6:**
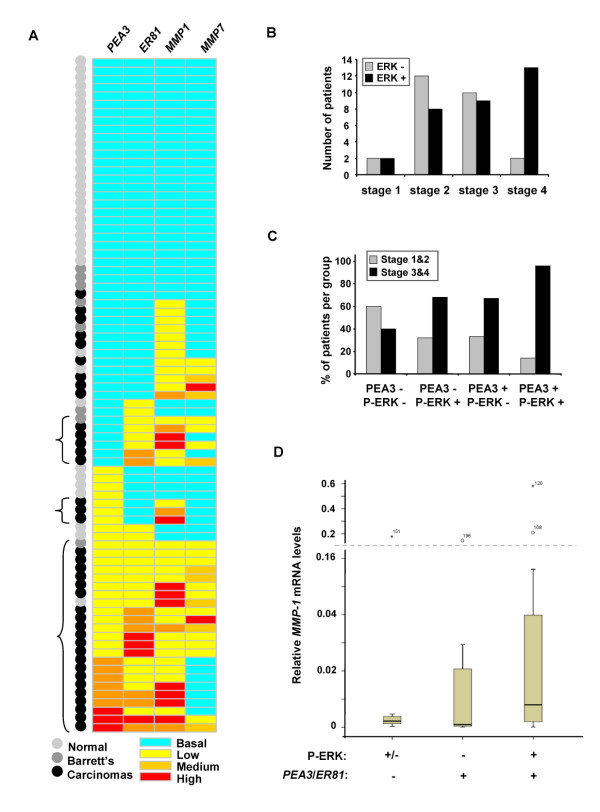
**Correlative expression of PEA3, ER81, *MMP-1 *with high levels of ERK signaling in oesophageal tissue specimens**. (A) Heat map of the relative mRNA levels of *PEA3*, *ER81*, *MMP1 *and *MMP7 *in the oesophageal tissue samples. Samples are categorized as "normal" (light grey dots) or from patients with Barrett's disease (dark grey dots) or oesophageal adnenocarcinomas (black dots). Expression was defined as mRNA levels more than one standard deviation above the mean for the normal samples for each gene. Basal levels are anything below this value (turquoise rectangles). Expression was then divided into three categories relative to the mean for the normal samples; high expression was anything more than 100 fold (red rectangles), medium was between 25-100 fold (orange rectangles) and low was up to 25 fold (yellow rectangles) over the mean. The primary data for individual samples is shown in additional file 1: figures S1 and S2. Groups of samples showing increased expression of *MMP-1 *and either *PEA3 *or *ER81 *relative to basal levels are bracketed. (B and C) Histograms correlating the number of patients with AJCC stage 1, 2, 3 and 4 disease and phospho-ERK (P-ERK) and PEA3 protein levels. (B) The number of patient samples showing the presence or absence of P-ERK is correlated with each disease stage. Positive ERK MAP kinase signalling is defined as more than 5% tumour cells staining positive for P-ERK at intensity 3-4. (C) Patients with a combination of high and low P-ERK levels and high and low PEA3 protein expression are correlated with either early stage disease (1&2; grey bars) or late stage disease (3&4; black bars). High PEA3 protein is defined as more than 5% tumour cells staining positive at intensity 4. Data are presented as the percentage of patients within each group representing the indicated combinations of P-ERK and PEA3 levels. (D) Box plots of *MMP1 *mRNA expression in oesophageal tissue taken from oesophageal adenocarcinoma patients. The data are grouped according to the presence or absence of high P-ERK levels and/or increased levels of *PEA3 *and/or *ER81 *(defined as mRNA levels more than two standard deviations above the mean for the normal samples for each gene). Median relative expression levels of *MMP-1 *are indicated for each combination of P-ERK and *PEA3*/*ER81*. mRNA expression is calculated relative to 18S ribosomal RNA. The box plot represents the inter-quartile range and the median value is indicated by the horizontal line. The y axes are split (indicated by dashed lines) and the high outliers are labelled by case number.

We have demonstrated that PEA3 family members control *MMP-1 *expression in oesophageal cancer cells. To establish whether PEA3 subfamily members might also play a role in controlling *MMP *expression in human cancers, we determined the levels of *MMP-1 *and *MMP-7 *mRNA expression in tissue samples from patients with oesophageal adenocarcinomas (Additional file 1: Figure S2). The majority of adenocarcinomas showed enhanced levels of *MMP-1 *(Additional file 1: Figure S2A) and/or *MMP-7 *(Additional file 1: Figure S2B) whereas only a few samples from normal oesophageal epithelium or from patients with Barrett's metaplasia showed enhanced levels of expression of either *MMP*. The data were then compared to the expression of *PEA3 *and *ER81 *in the same samples (Figure 6A). There is a clear clustering of samples which express enhanced levels of either *PEA3*, *ER81 *or both and the expression of *MMP-1*. In many cases, *MMP-7 *is also overexpressed at the same time as *PEA3 *and/or *ER81*, although the correlation is not as tight. This is consistent with our findings in oesophageal cell lines where links between PEA3 subfamily members and *MMP-7 *expression were not readily apparent. Importantly, the majority of samples that showed increased levels of both a PEA3 family member and *MMP-1 *were derived from adenocarcinomas.

ERK MAP kinase signaling is an important driver of PEA3-mediated transactivation and downstream *MMP-1 *expression in oesophageal adenocarcinoma-derived cell lines. We therefore also investigated the status of ERK pathway activation by monitoring the levels of the active phosphorylated form of ERK (P-ERK) using the TMAs containing samples from patients with adenocarcinomas. Samples were then scored as P-ERK positive if more than 5% tumour cells stained positive for P-ERK at intensity 3-4. Samples were then grouped according to whether they were derived from patients with AJCC stage 1, 2, 3 and 4 disease and the P-ERK status recorded (Figure 6B). Whereas early stage tumours show little preference for P-ERK positivity, stage 4 samples are predominantly positive for P-ERK, suggesting a correlation with more advanced disease. We also investigated whether the presence of both high PEA3 protein and P-ERK levels would correlate with disease severity (Figure 6C). While high levels of either PEA3 or P-ERK alone show only moderate association with later stage tumour samples, there is a clear over-representation of high levels of both P-ERK and PEA3 with late stage tumours. As stage 3 and 4 represent metastatic stages, this is in keeping with a role for PEA3 in promoting metastasis in response to ERK pathway signaling. We therefore examined whether P-ERK levels and PEA3 subfamily expression in adenocarcinoma samples might correlate with the expression of a key driver of metastasis, *MMP-1*. There is a general trend indicating enhanced expression of *MMP-1 *in the presence of either enhanced *PEA3 *and/or *ER81 *mRNA alone and this is further increased in samples exhibiting concomitant increased P-ERK levels (Figure 6C), although due to small sample sizes, these values did not reach statistical significance.

Together these data therefore show a clear correlation between PEA3 subfamily member expression and the expression of *MMP*s in adenocarcinoma tissue samples. Furthermore, enhanced levels of ERK pathway signaling combined with PEA3 expression correlate with advanced metastatic disease. Thus, the ERK-PEA3-MMP-1 axis which functions in oesophageal adenocarcinoma cell lines appears to also be operative in human oesophageal cancer.

## Discussion

The PEA3 subfamily of ETS-domain transcription factors have been shown to be important drivers of cancer cell metastasis, which is best studied in breast cancers [14]. Here we show that PEA3 subfamily members are overexpressed in oesophageal adenocarcinomas and promote cell proliferation and invasion in oesophageal cancer-derived cell lines. *MMP-1 *is identified as an important target for PEA3 subfamily members in cell line models and is co-expressed with these transcription factors in human adenocarcinomas. Furthermore ERK pathway signalling plays a critical positive role in PEA3-driven processes in cell lines and enhanced levels are also prevalent in advanced stage adenocarcinomas. Our data therefore demonstrate a broader role for the ERK-PEA3-MMP-1 axis in tumourigenesis and identify it as a potentially important component in adenocarcinoma development and progression.

Our results point to a role for PEA3 subfamily members in driving invasion, one of the key transformations that occur during tumour metastasis. In oesophageal adenocarcinoma-derived OE33 cells, depletion of PEA3 leads to a reduction in the expression of *MMP-1*, an important player in metastasis (Figure 2) and reduced invasion (Figure 4). While PEA3 appears to play an important role in controlling these processes, we cannot rule out a contributory role for the PEA3 subfamily member ER81, as depletion of PEA3 leads to reductions in *ER81 *levels (Figure 2). Moreover, it is firmly established that the ERK pathway leads to PEA3 family activation [30,31], and in keeping with this observation, inhibition of ERK signalling blocks invasion and reduces *MMP-1 *expression in OE33 cells (Figure 5). Importantly, these cells exhibit high levels of basal ERK pathway signalling in the absence of mitogenic stimulation (Figure 5A). In contrast, Flo1 cells contain little *MMP-1 *mRNA or protein and very low levels of phospho-ERK (Figure 5A) despite high levels of ER81 and PEA3 (Figure 2A) which suggests that the lack of ERK pathway signalling might be the reason for the lack of MMP-1 expression in these cells. Indeed, activation of the ERK pathway in Flo1 cells promotes MMP-1 expression. Thus OE33 cells appear to have been rewired to cause constitutive high levels of ERK signalling, to express high levels of *PEA3 *and *ER81 *and hence to have high levels of MMP-1 which can help drive cell invasion.

The relationship between PEA3 and ER81 and target gene expression is not entirely clear. These two proteins share considerable sequence homology and have a conserved domain structure, including an almost identical DNA binding domain. Thus target gene selection and activation are likely to proceed in a similar manner. Interestingly, depletion of ER81 also causes reductions in *MMP-1 *levels. However, depletion of ER81 also causes reductions in *PEA3 *mRNA levels hinting at potential cross-regulation. This is even more pronounced in the reciprocal direction where depletion of PEA3 leads to substantial decreases in ER81 levels. This is unlikely to be a non-specific effect or chance cross-hybridisation as four different PEA3 siRNAs cause reductions in ER81 expression (Figure 2F). This suggests that there might be reciprocal cross-regulation of ER81 and PEA3 on each others' expression. Indeed, the upstream ERK pathway that activates ER81 and PEA3 transactivation capacity is also important for the expression of both ER81 and PEA3. Further studies are needed to support this model for mutual cross regulation which might reinforce the expression levels of each transcription factor. However, the current data suggests an important role for PEA3 and/or ER81 in promoting *MMP-1 *expression and subsequent invasion.

A major finding from our work is that PEA3 is also important for promoting OE33 cell proliferation. Again, ERK pathway signalling also has a crucial function in this context. Additional work is required to determine the molecular basis to PEA3-driven oesophageal cancer cell proliferation but MMP-1 expression is unlikely to account for the altered proliferation as PEA3 siRNA construct B does not significantly reduce *MMP-1 *levels (Figure 2G) but it does profoundly affects proliferation (Figure 4E). A previous study in breast cancer cells suggested a role for PEA3 in proliferation control as it was shown that PEA3 regulates *Cyclin D3 *expression, a key regulator of the cell cycle and affects cell cycle progression [39]. Moreover, in p53-depleted ovarian cancer cells, PEA3 has been shown to regulate the p21, a potent inhibitor of the cell cycle [40]. It is likely that the expression or activity of key cell cycle regulators such as cyclin-CDK complexes or their inhibitors are either directly or indirectly controlled by PEA3 subfamily members in oesophageal adenocarcinoma cells.

To provide evidence for the existence of the same regulatory pathway in human adenocarcinoma samples, the levels of *PEA3*, *ER81*, *MMP-1 *and the activation of the ERK pathway were monitored. There was a clear co-upregulation of *PEA3 *and *ER81 *with *MMP-1 *and, to a lesser extent, *MMP-7 *in adenocarcinoma samples (Figure 6B), suggesting a causative role for PEA3 subfamily members in driving *MMP-1 *expression. Importantly, high levels of PEA3 protein expression correlated with N stage disease (Figure 1C), and a combination of high PEA3 levels and high ERK activation correlated with late stage metastatic forms of the disease (Figure 6C). Thus, enhanced PEA3 levels coincide with molecular markers of metastasis such as *MMP-1 *and are found in the more advanced metastatic stages of the disease. While these data are correlative, they are consistent with our work in oesophageal adenocarcinoma-derived cell lines and indicate that the ERK-PEA3-MMP-1 axis likely plays an important role in driving the progression of oesophageal adenocarcinomas in humans. Importantly we find little evidence to support a role for the ERK-PEA3-MMP-1 axis in samples from patients with Barrett's metaplasia which is thought to be a forerunner to the formation of oesophageal adenocarcinomas and hence potentially represents an early stage of the disease. Low expression levels of PEA3 subfamily members and relatively low levels of MMPs are observed Barrett's metaplasia samples (Additional file 1: Figures S1 and S2). We were unable to make meaningful comparisons between patient samples with Barrett's oesophagous and early stage 1 adenocarcinomas and hence the potential transition period, because to the paucity of samples in the latter class due to the tendency of patients to present with the disease once it has become firmly established. The activation status of the ERK-PEA3-MMP-1 axis does however represent a potentially attractive prognostic indicator of advanced oesophageal adenocarcinomas.

## Conclusions

In summary, this study shows that the ERK-PEA3-MMP-1 axis is upregulated in oesophageal adenocarcinoma cells where it plays a role in promoting invasion, and in the case of the ERK-PEA3 subpart, a role in enhancing proliferation. Components of the ERK-PEA3-MMP-1 axis are also upregulated or hyperactivated in adenocarcinoma samples indicating that this axis is a potentially important driver of the metastatic progression of oesophageal adenocarcinomas.

## Materials and methods

### Tissue collection

Ethical approval was granted by Wrightington Wigan and Leigh Ethics Committee, UK in 2004. Tissue was collected from 70 patients with oesophageal adenocarcinomas, 28 with Barrett's oesophagus and 55 healthy controls. Adenocarcinomas at the gastro-oesophageal junction were classified as oesophageal adenocarcinomas. Age and date at diagnosis, gender, co-morbidity, smoking status and survival was recorded. Details of the histological grade of tumour and stage, using the TNM and AJCC criteria were collected. Information on treatments including surgery, chemotherapy, radiotherapy and palliation were also recorded. Biopsy samples, approximately 4 mm in size, were taken at the time of endoscopic examination. Biopsy and surgical samples were rapidly frozen in liquid nitrogen and stored at -80°C until needed. Paraffin blocks were used to construct tissue microarrays for immunohistochemistry. Frozen biopsy and surgical samples were used for RNA extraction.

### Cell lines, cell culture and western analysis

OE33, and OE21 (oesophageal adenocarcinoma) cell lines (kindly provided by Caroline Hill, Cancer Research UK, LRI), Flo1 and Het1A oesophageal cells (kindly provided by Laura Hardy, Molecular Epidemiology Unit, Leeds), 293T and SW480 cells were all grown in DMEM (Invitrogen) medium except SW480 cells which were grown in RPMI (Invitrogen) medium. All the cell lines were grown with 10% foetal bovine serum (FBS) (Invitrogen) and penicillin (100 units/ml) and streptomycin (100 μg/ml) (P+S) (Invitrogen) at 37°C with 5% carbon dioxide. Cells were grown with 10 nM PMA, 10 mM U0126 (Sigma^®^) or the carrier solvent DMSO (Sigma^®^) when indicated. Cell lysis was carried out as previously described [36]. For western analysis, 100 μg of cell lysate was typically used for SDS-PAGE. Following transfer to a nitrocellulose membrane proteins were detected with either ERK2 (New England Biolabs), pERK (New England Biolabs), MMP-1 (Abcam Ab38929) or MMP-7 (Santa Cruz Sc-8832) antibodies.

### RNA isolation and RT-PCR analysis

RNA was extracted using RNeasy (Qiagen) according to the manufacturer's protocol. Tissue specimens were additionally treated with DNase I (Qiagen) to remove DNA contamination. RNA integrity was confirmed in tissue specimens with a 2100 Bioanalyser with a RNA 6000 Nano Assay Lab Chip^® ^kit (Agilent Technologies, US). Only specimens with a RIN > 5 were analysed further. Sybr Green RT-PCR (for real time RT-PCR) and single step RT-PCR (for semi-quantitative standard RT-PCR) kits (Qiagen) were utilised according to the manufacturer's protocol. The primers used were PEA3, ADS2679 (5'-GGACTTCGCCTACGACTCAG-3') and ADS2680 (5'-CGCAGAGGTTTCTCATAGCC-3'); ER81, ADS2681 (5'-TCCCTCCATCGCAGTCCATA-3' and ADS2682 (5'-GGAAAGCTTTGGCTGGCCG-3'); MMP-1, ADS2669 (5'-GGTCTCTGAGGGTCAAGCAG-3') and ADS2670 (5'-AGTTCATGAGCTGCAACACG-3'); MMP-7, ADS2675 (5'-CCAAATCAACCATAGGTCCA-3') and ADS2676 (5'-TTGAGATAGTCCTGAGCCTG-3') (for single step RT-PCR); ADS2671 (5'-GAGTGCCAGATGTTGCAGAA-3') and ADS2672 (5'-AAATGCAGGGGGATCTCTTT-3') (for real-time RT-PCR); 18S, ADS4005 (5'-CGGCTACCACATCCAAGGAA-3') and ADS 4006 (5'-GCTGGAATTACCGCGGCT-3'); Osteopontin, ADS2673 (5'-TTGCAGTGATTTGCTTTTGC-3') and ADS2674 (GTCATGGCTTTCGTTGGACT-3'); VEGF ADS2678 (5'-AAGTGGTCCCAGGCTGCA-3') and ADS2679 (5'-ACTCCAGGCCCTCGTCA-3'); GAPDH, ADS2184 (5'-ACAGTCAGCCGCATCTTCTT-3') and ADS2185 (5'-TTGATTTTGGAGGGATCTCG-3'). Real time PCR reactions were run on a Rotor Gene RG-3000 (Corbett) and analysed with Rotor-Gene 6 software. Data are presented relative to 18S RNA levels in the same samples. For relative comparison of mRNA levels from tissue specimens, data were further normalized to the level of each gene in a standard concentration of RNA isolated from OE33 (for *MMP-1 *and *MMP-7*), SW480 (for *PEA3*) and Flo1 (for *ER81*) cells.

### Immunohistochemistry

Tissue microarray blocks were constructed from surgical resection tumour blocks and biopsies as follows; three 0.6 mm cores were taken from each tumour using a precision arraying instrument (Beecher Instruments). These cores were then arrayed into a new "recipient block". Sections (4 μm thick) were cut with a microtome from each TMA and mounted on adhesive slides (Vision BioSystems™). One H&E stained slide was made to use as a reference for the cores. Three arrays were constructed for each case and stained with PEA3 (Santa Cruz Sc 113) and pERK antibodies (New England Biolabs #437S) at a 1:20 and 1:100 dilution respectively. A negative control slide was tested without the primary antibody to detect any background staining or false positive results. Three cores for each specimen were constructed and scored by two histopathologists blinded to the clinical details. A positive score was determined by the presence of positive staining in 5% of tumour cells. An intensity score of 1-4 was also determined. Moderate to high expression (intensity score 3 and 4) was judged to be present if staining was visible easily at ×20 magnification. The highest score in the triplet of cores was recorded. We took moderate to high expression as positive for PEA3 protein expression.

### Invasion assays

2 × 10^5 ^cells were seeded on the upper, serum free, 8 μm Matrigel chamber and allowed to migrate to a lower chamber containing 10% FBS. After 24-48 hours, the upper surface was cleaned with a cotton bud. Cells on the lower surface were fixed with 4% paraformaldehyde (Sigma) and stained with 0.5% Crystal violet (Sigma). Cells were counted in 10 fields at ×10 magnification, the highest scoring outlier field was omitted and then the average numbers per field from the remaining 9 fields was calculated. The data are presented relative to a control condition for each experiment. Each experiment was repeated at least three times.

### Proliferation assays

Cells that did not stain with Trypan Blue 0.4% (Sigma) were termed viable. 1-2 × 10^5 ^viable cells were grown for 96 hours. Adherent cells were detached using 200 μl Trypsin 0.05% (Invitrogen). Viable and non viable cells were counted at 24 hour intervals using a haemocytometer.

### siRNA and plasmid transfection

Short interfering (si) RNAs directed against human PEA3, ER81, MMP-1 (SMARTpools; Dharmacon), PEA3 (individual deconvoluted SMARTpool constructs; Dharmacon) and a non-targeting scrambled sequence (Santa Cruz) were used. Lipofectamine RNAiMAX (Invitrogen) was used for siRNA transfection according to the manufacturer's protocols. Lipofectamine 2000 (Invitrogen) was used for DNA transfection or combined siRNA and DNA transfection according to the manufacturer's protocol. The final concentration of siRNAs was 10 nM and the media was replaced after 4-24 hours. The cells were allowed to grow for a further 24 to 96 hours after transfection.

### Luciferase reporter assays

For reporter gene assays, 15 × 10^4 ^cells were plated in each well of a 6 well plate and transfected with vectors encoding MMP-1-luciferase (pColI-luc containing the MMP-1 promoter -517/+63) (pAS2701; kindly provided by Olivier Kassel [42]) (500 ng), pCH110 (100 ng) and either PEA3 (pAS1801 [36]) (500 ng) or empty pCDNA3 vector (500 ng). 10 nM siRNA was also added to the cells. After 48 hours the cells were washed, lysed and luciferase and β-galactosidase activities determined according to the kit manufacturer's instructions (Tropix) using a TD-20/20 luminometer (Turner Designs). The luciferase activity for each sample relative to β-galactosidase activity was then calculated.

## Competing interests

The authors declare that they have no competing interests.

## Authors' contributions

RK contributed to the study design, conducted the majority of the experiments, and helped with manuscript preparation. BG contributed towards experimental design. PD and CG performed the TMA experiments and data analysis. AY provided clinical training, contributed to study design and coordination and data interpretation. ADS contributed to experimental design and coordination and wrote the manuscript. All authors read and approved the final manuscript.

## Supplementary Material

Additional file 1 **Figure S1. mRNA expression levels of *PEA3 *and *ER81 *in oesophageal tissue**. (A and B) mRNA levels of *PEA3 *(A) and *ER81 *(B) relative to 18S RNA in tissue specimens are presented. All samples were standardised to expression in SW480 (for *PEA3*) and Flo1 (for *ER81*) cell lines and are presented on a log_2 _scale. The average relative mRNA levels and standard deviations derived from at least two readings from one sample are shown. The individual tissue specimens are numbered. The samples are grouped according to the indicated oesophageal tissue sub-types. The average gene expression in each category is shown in red. They axis is split for both genes. **Figure S2. mRNA expression levels of *MMP-1 and MMP-7 *in oesophageal tissue**. (A and B) mRNA levels of *MMP-1 *(A) and *MMP-7 *(B) relative to 18S RNA in tissue specimens are presented. All samples are standardised to expression in OE33 cells and are presented on a log_2 _scale. The average relative mRNA levels and standard deviations derived from at least two readings from one sample are shown. The individual tissue specimens are numbered. The samples are grouped according to the indicated oesophageal tissue sub-types. The average gene expression in each category is shown in red. They axis is split for both genes. Click here for file
