# Supplementary figures and images for: The ERK MAP kinase-PEA3/ETV4-MMP-1 axis is operative in oesophageal adenocarcinoma
Source: Mol Cancer. 2010 Dec 9;9:313. doi: 10.1186/1476-4598-9-313 (PMC3009708; doi:10.1186/1476-4598-9-313)

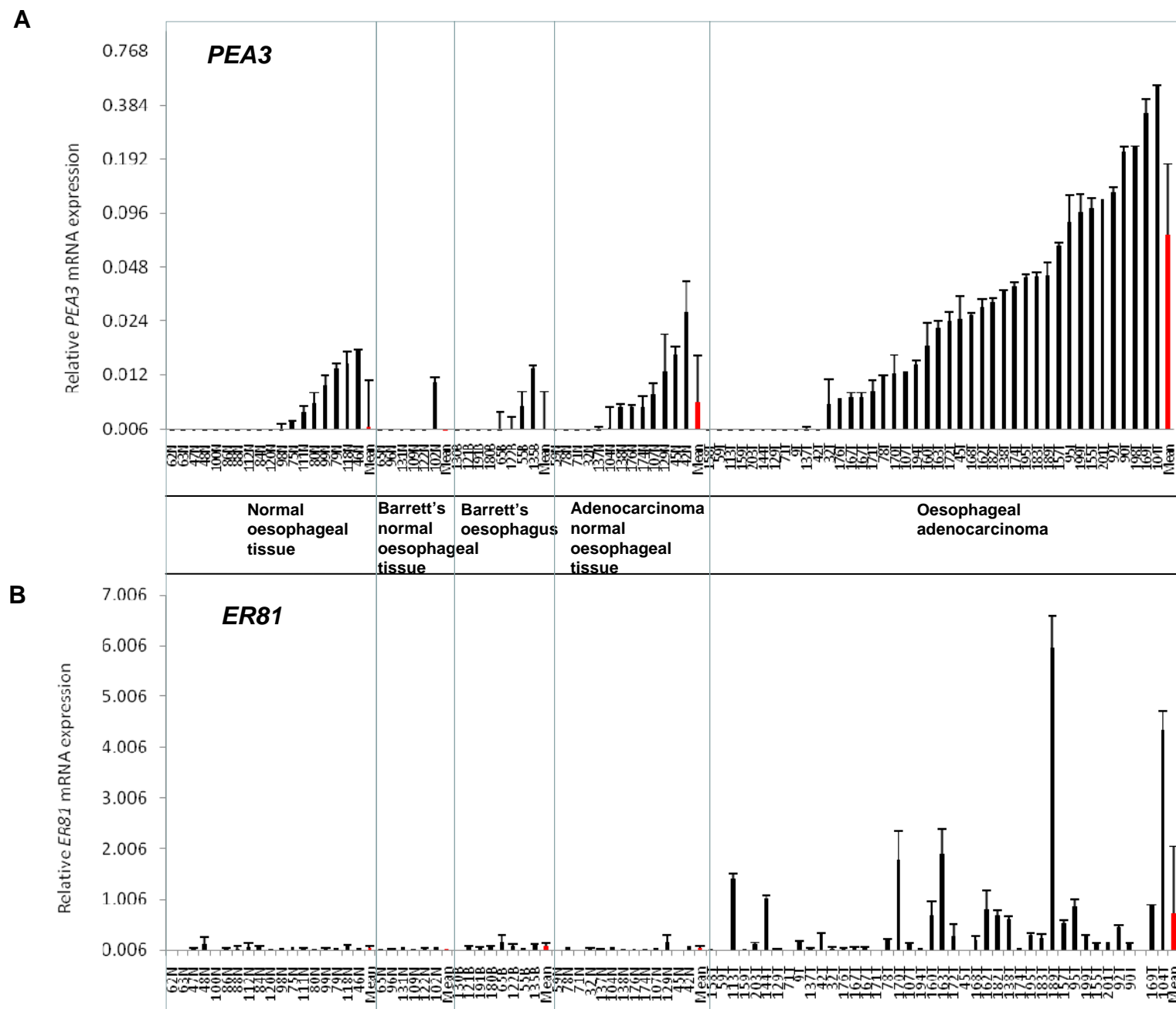

**Fig. S1**

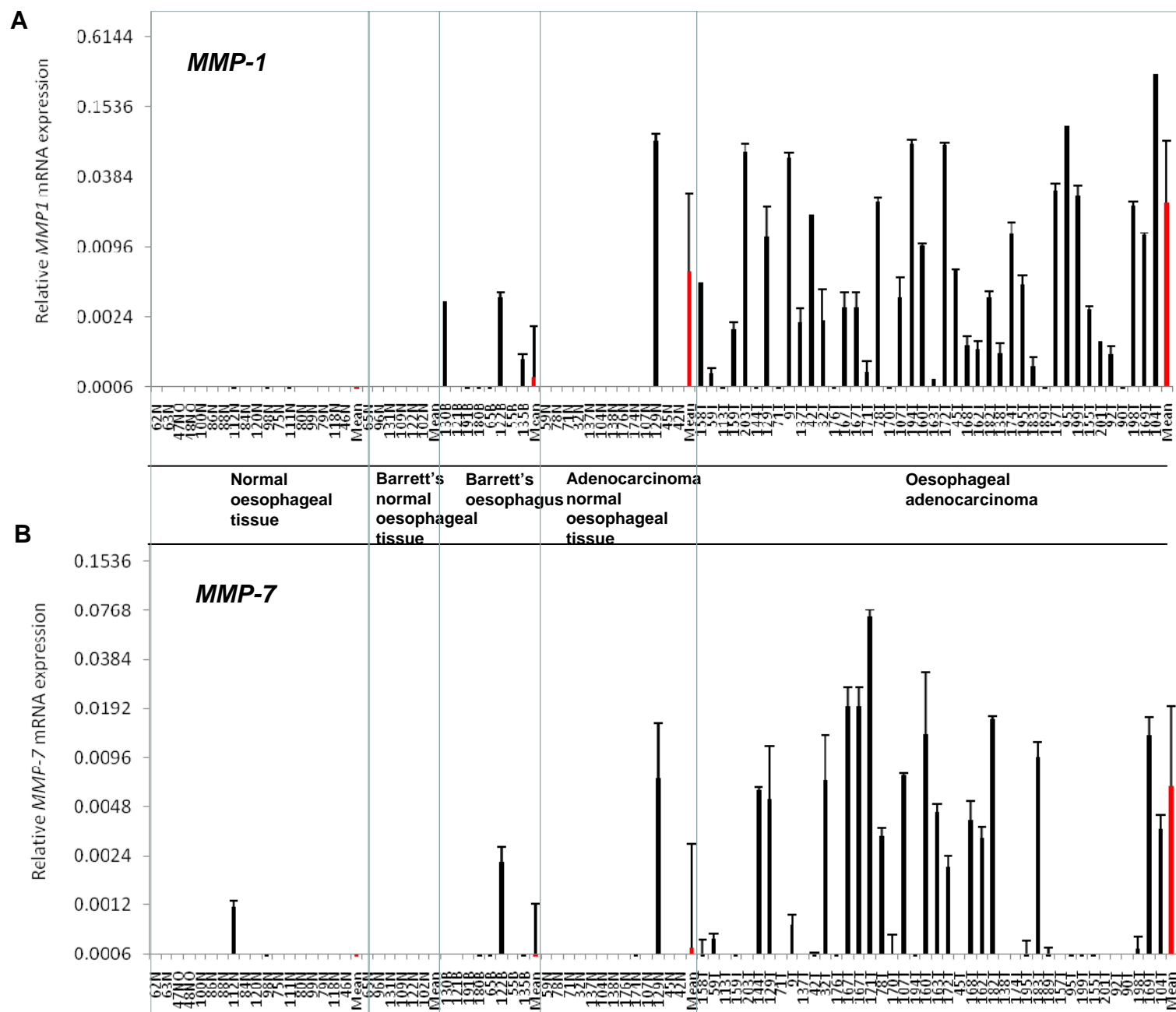

**Fig. S2**

Supplement: Additional file 1 — Figure S1. mRNA expression levels of PEA3 and ER81 in oesophageal tissue. (A and B) mRNA levels of PEA3 (A) and ER81 (B) relative to 18S RNA in tissue specimens are presented. All samples were standardised to expression in SW480 (for PEA3) and Flo1 (for ER81) cell lines and are presented on a log2 scale. The average relative mRNA levels and standard deviations derived from at least two readings from one sample are shown. The individual tissue specimens are numbered. The samples are grouped according to the indicated oesophageal tissue sub-types. The average gene expression in each category is shown in red. They axis is split for both genes. Figure S2. mRNA expression levels of MMP-1 and MMP-7 in oesophageal tissue. (A and B) mRNA levels of MMP-1 (A) and MMP-7 (B) relative to 18S RNA in tissue specimens are presented. All samples are standardised to expression in OE33 cells and are presented on a log2 scale. The average relative mRNA levels and standard deviations derived from at least two readings from one sample are shown. The individual tissue specimens are numbered. The samples are grouped according to the indicated oesophageal tissue sub-types. The average gene expression in each category is shown in red. They axis is split for both genes. [file 1476-4598-9-313-S1.PDF]
